# The calculator analogy: Epistemic virtues for using LLMs

**DOI:** 10.1016/j.techsoc.2025.103198

**Published:** 2026-04

**Authors:** Cristina Voinea, Sebastian Porsdam Mann, Julian Savulescu, Brian D. Earp

**Affiliations:** a Uehiro Oxford Institute, University of Oxford, LittleGate House, 16-17 Saint Ebbe’s St, Oxford, OX1 1PT, UK; b Centre for Advanced Studies in Bioscience Innovation Law, University of Copenhagen, Denmark; c Faculty of Law, University of Oxford, UK; d Centre for Biomedical Ethics, Yong Loo Lin School of Medicine, National University of Singapore, Singapore, Singapore

**Keywords:** Large language models (LLMs), Calculator analogy, Technological affordances, Epistemic virtues, Educational technology, Human–AI interaction

## Abstract

Like the arrival of calculators in 1970s classrooms, large language models (LLMs) provoke both fears of intellectual deskilling and hopes of more efficient learning. In this paper we analyze the calculator analogy, arguing that while it is a useful starting point to understand the potential impact of LLMs in education, it is ultimately insufficient. We show where the analogy holds and, just as importantly, where its limitations reveal the unique pedagogical challenges posed by LLMs. These challenges arise from fundamental differences in how calculators and LLMs mediate learning, reflecting the distinct affordances of each technology. We argue that because of their affordances, realizing the educational potential of LLMs calls for cultivating epistemic virtues suited to human–AI interaction, such as patience, reflective engagement, or intellectual vigilance and humility. Equally, LLM design must actively foster these virtues through features like built-in prompts, feedback loops or reflective questions, to name just a few.

## 1 Introduction

The arrival of large language models (LLMs) in classrooms has sparked heated debates (Pham, 2025). At one extreme are those who foresee an “educational apocalypse” (Ceres, 2023). They warn that “AI integration will reward prompting over genuine reasoning skills” (Cook, 2025) and that students are “outsourcing thought to ChatGPT” (Spirlet, 2025), which will lead to a loss of abilities to reason or write independently (Anderson et al., 2025). Ultimately, LLMs will contribute substantively to “the collapse of public education” (Ko, 2024).

At the other extreme are those who see LLMs as tools that can help everyone learn better, faster and more efficiently. The main hope is that LLMs will allow students and professors alike to build a “personalized tutor in every subject” (Reporter, 2025), which can adapt to students’ interests, educational needs and learning pace. LLMs could also be used as “powerful brainstorming tools” (Bushweller, 2025) and could “summarize large stretches of text which could save students and teachers time and help them to instead focus on discussion and learning” (Extance, 2023). In the long run, it is believed that LLMs will “help usher in a future where everyone has access to a world-class education” (Gates, 2024).

These polarized perspectives might suggest that we’re confronting a completely new phenomenon. Yet more than fifty years ago, in the 1970s, pocket calculators provoked similar disputes. On the one hand, some feared that calculators would lead to the loss of basic skills, such as an intuitive grasp of numbers, procedural fluency, and the kind of understanding that develops only through direct engagement with mathematical problems (Pendleton, 1975; Sheets, 2007). On the other hand, reformers insisted that calculators promised to relieve students of the tedium of calculation, which was hoped to create more time to engage in higher-order thinking (Ellington, 2003).

In fact, this parallel has not escaped public attention, leading some to draw an analogy between calculators and LLMs. Tech writers have described LLMs as “calculators for words” (Vijayan, 2023; Jorgenson, 2025); certain educators have suggested that they should become “like calculators for writing” (Ceres, 2023); and one group of researchers has called AI “the calculator of the twenty-first century classroom” (Anderson et al., 2025). Even the CEO of Open.AI, Sam Altman, has pushed for the calculator analogy, saying “We adapted to calculators and changed what we tested for in math class, I imagine. This is a more extreme version of that, no doubt, but also the benefits of it are more extreme, as well” (quoted in Mok, 2023). In short, according to this perspective, LLMs are not fundamentally different from calculators when it comes to their net benefits in the classroom.

In this paper we revisit the calculator analogy. While it offers a useful starting point to think about the effects of LLMs in classrooms, we argue that it is ultimately limited: calculators and language models afford fundamentally different kinds of engagement. Attending to this difference helps us identify pedagogical challenges specific to LLMs. We argue that using LLMs to support rather than replace learning requires cultivating epistemic virtues, such as patience, reflective engagement, intellectual vigilance and humility, and designing LLMs in ways that actively foster these virtues.

We start by unpacking why the calculator analogy is tempting. Next, we revisit the calculator debates of the 1970s, showing that the impact of calculators was shaped less by the device itself than by the pedagogies into which it was embedded. We then turn to emerging evidence on LLMs’ impact on students, identifying both benefits and risks. With all this in mind, we zoom in on the calculator analogy once again, showing where it proves inadequate to capture the full scope of LLMs’ likely impact. Here we draw on the notion of technological affordances to explain why LLMs, unlike calculators, can invite students to bypass the very processes of thinking that education is meant to cultivate. Finally, we argue that realizing the educational potential of LLMs requires both cultivating specific epistemic virtues and designing systems that actively support those virtues. The conclusion pulls all of these strings together, noting that although education has always sought to nurture reflective and critical thinkers, the presence of LLMs transforms what such reflection and critique demand.

## 2 The calculator analogy

As allusions to the history of calculators accumulate in search of clues about how to handle LLMs, it is worth asking whether the comparison really makes sense. There are several reasons which, on their face, challenge the relevance and fit of the analogy. Calculators manipulate numbers through deterministic algorithms; enter the same input twice, and you get the same output. LLMs, by contrast, generate language through sampling from distributions over tokens; as a result, very few outputs are exactly alike. Further, calculators operate within closed symbolic systems governed by formal mathematical rules, wherein every operation has a single, verifiable outcome. LLMs, by contrast, work in open semantic spaces, modeling patterns in how words appear together to generate language that fits the context, instead of relying on set grammatical structures. At first glance, then, calculators and LLMs appear to share little in common beyond being tools used for learning. Their underlying logics, domains of operation, and outputs differ so much that the analogy might seem misplaced.

But there are some aspects of both technologies that are indeed analogous, and instructively so. Both can detract from, or support, genuine learning depending on how they are used. Both reorganize the process of thinking (albeit, in different ways). In either case, educational outcomes depend less on the tool’s internal mechanisms than on how, specifically, students—with the support of their teachers and mentors—integrate it into their reasoning processes.

To see this parallel more clearly, we can frame learning with technology as a flow of information across three stages: inputs, transformation, and outputs. The student provides inputs to the tool, whether a calculator or an LLM: these may take the form of prompts, numerical values, logical assumptions, or problem formulations. The tool performs a transformation on those inputs. The student must then interpret and verify the outputs. For learning to occur, students must retain some degree of agency and responsibility at one or more stages. When any stage becomes fully automated or bypassed, the process shifts towards the delegation of mental processes (such as recall, reasoning, or judgment) to the tool.

This suggests that each technology redistributes cognitive labour, and each can either support or hamper learning depending on how cognitively engaged students are when using them. Yet the analogy also has limits, which become evident when we analyze these technologies through the lens of their *affordances*. Before examining where the analogy breaks down, though, it’s worth understanding what actually happened when calculators arrived in classrooms. What did five decades of research reveal about when (partial) automation supports or undermines learning?

## 3 The calculator precedent

Calculators did not emerge in a vacuum. They followed a long succession of mathematical aids such as the abacus, logarithmic tables, and slide rules, each of which provoked familiar fears about dependency and deskilling in mental arithmetic. As with these earlier mathematical aids, the arrival of calculators in classrooms raised fears of cognitive deskilling.

Contemporary reporting showed that some teachers feared early calculator use would “make pencil-and-paper math obsolete” and might even leave children unable to “count if their calculator batteries ever go dead” (Pendleton, 1975). Others, though, insisted that calculators could free students from tedious computation and allow them to focus on problem solving and conceptual understanding (Suydam, 1976; see also Monaghan, 2016). What made calculators distinctive was not the nature of these anxieties but their scale and the systematic empirical study they eventually generated, making them the first well-documented test case of how automation of previously human-only cognitive tasks affects learning when deployed broadly.

The debates that followed led to half a century of research, the results of which have transformed those anxieties into greater understanding of how automation interacts with learning. Studies converge on five basic insights.

● *First*, calculator use generally maintains or modestly improves mathematical learning, with no evidence of *global* skill erosion across grades or ability levels. Across four meta-analyses examining various types of calculator and broader computer use in mathematics instruction, average effects on procedural, computational, conceptual, and problem-solving measures were small to moderate and largely positive (Hembree & Dessart 1986; Ellington, 2003, 2006; Li & Ma, 2010).
● *Second*, the strongest gains occur when calculators are deliberately and actively integrated into the pedagogical approach, that is, used for exploration, conceptual understanding, or within specially designed curricula, rather than applied functionally for drills or checking of results. Studies that embedded calculators in what researchers called ‘special instruction’, that is, curricula explicitly designed around the technology, reported substantially larger improvements than other studies (Hembree & Dessart 1986; Ellington, 2003).
● *Third*, whether there is alignment between the use of calculators in instruction and assessment settings makes a large difference to observed effects. Performance appears to improve most when the conditions of testing match the mode of learning, and remains flat or shows only small benefits when calculators are used in instruction but excluded from tests or exams. Students who practiced with calculators but were examined without them showed minimal gains (but, importantly, also no detriments to learning), while those who used calculators in both contexts demonstrated consistent improvements across operational, computational, conceptual, and problem-solving skills (Hembree & Dessart 1986; Ellington, 2003, 2006).
● *Fourth*, attitudes toward mathematics consistently improve when students are allowed to use calculators. Students reported greater confidence and enjoyment related to mathematics and no rise in anxiety, compared to students not allowed to use calculators. This affective shift appeared across calculator types and educational levels, suggesting that appropriate use of technology can enhance motivation and curiosity rather than dulling them (Hembree & Dessart 1986; Ellington, 2003, 2006; Li & Ma, 2010).
● *Fifth*, beyond effects on individual learning and motivation, the calculator debates revealed that technological innovations also raise questions of educational equity—showing that who benefits depends less on access to devices than on access to effective pedagogy. While some studies found substantial benefits particularly for special-needs students when technology was carefully designed to support their learning, differences in outcomes across student groups more consistently reflected differences in instructional quality, not mere access to technology (Li & Ma, 2010). Schools and teachers able to integrate technology conceptually, to align it with curriculum and assessment, and to provide sustained pedagogical support derived the greatest and most equitable benefits. In well-resourced environments, calculators became platforms for conceptual exploration and modeling of mathematical ideas; in under-resourced ones, they risked becoming something more akin to computational shortcuts. The calculator era thus showed that technological innovation magnifies existing pedagogical strengths and weaknesses rather than erasing them, and that the equity effects of any educational technology flow as much from how it is embedded in practice as from the features of the device itself.

Taken together, these findings point towards the conclusion that the effects of calculators on students depended to a great extent on *how* they are used and the educational contexts in which they are embedded.

## 4 From calculators to LLMs

The calculator case suggests that much depends on pedagogy and context. In the case of LLMs, however, the technology’s affordances also play a crucial role in shaping learning outcomes. Before turning to these affordances in detail, we briefly review emerging evidence on the benefits and risks of LLMs in education.

### 4.1 Emerging evidence: conditional benefits and risks

LLMs haven’t been with us for too long, so we don’t yet have longitudinal studies on their long-term effects. The literature is young and methodologically heterogeneous, and its findings should be treated as provisional rather than definitive. Nonetheless, the available studies suggest that LLMs likely have similar kinds of effects as earlier technologies, but with both greater opportunities and graver risks.^1^

As with calculators, four large-scale meta-analyses published in 2025 converge on a generally positive but nuanced picture (Deng et al., 2024; Huang et al., 2025; Liu et al., 2025; Wang & Fan, 2025). In all four studies, LLM use tends to be associated with moderate to large effects on achievement and knowledge acquisition, moderate but positive effects on affect and motivation, and moderate positive effects on higher-order reasoning and learner autonomy. Huang and colleagues’ (2025) analysis found the strongest effects for *qualification*, that is, the acquisition of knowledge and skills, while effects on *subjectification*, the development of autonomy and agency, were smaller and more fragile. These results come with important caveats. Most interventions were brief, often lasting only weeks, and lacked follow-up assessments. Measures varied widely across studies, as did implementation methods, making it difficult to disentangle tool effects from pedagogical effects.

The empirical pattern suggests that outcomes depend on whether LLMs are used to extend or bypass reasoning. When students interact with these systems iteratively through prompting, interrogating outputs, and revising successive drafts, the technology appears to have more positive effects. Lu and colleagues (2024) found that when students used AI-generated drafts as a starting point and then revised them, they showed stronger critical thinking and more confidence in their abilities than students who wrote their papers completely on their own. Kasneci and colleagues (2023) observed that students using LLMs for brain-storming generated broader initial ideas, though they struggled to develop depth without further guidance. These preliminary findings suggest that LLMs, like calculators, are instruments whose value depends on how they are introduced, taught, and used. Use of iterative, structured, and explicit prompts to critique and revise draft material appears capable of bringing about more engagement and reflection than rote use in which prompts and outputs are copied and pasted.

But this is only part of the story. To understand why, we need to look beyond outcomes, context, and pedagogy to the concept of *affordances*, the possibilities for action that a technology invites or constrains. It is through their affordances that tools shape not only what students do, but how learning itself unfolds. We turn to this problem next.

### 4.2 What the analogy misses: different technological affordances

Affordances are the activities and practices a specific technology makes possible or constrains, under certain circumstances (Gibson, 1966, 1979; Klenk, 2021). But, very importantly, affordances are not only possibilities for action, but also significant influences on the behaviour of agents interacting with that artefact, with the potential to shape how users think, act, and make sense of tasks (Withagen et al., 2012).

Consider a spellchecker software, whose basic affordance is that it allows users to spot and fix spelling errors. But, when used for long periods of time, a spellchecker might also shape behaviour: users could, in principle, become less likely to learn spellings explicitly, and more likely to rely on the red underline. Over time, this can change how they approach writing: they may focus less on accuracy while drafting, because they’ve learned the tool will “catch it later”. So, the more profound affordance of a spellchecker is not only that it will correct one’s spelling errors, but that it can also make users not think as carefully about spelling while they write. This is the sense in which affordances both enable and influence action.

In educational settings, affordances matter because they influence how cognitive labour is distributed between learner and tool. In other words, what the concept of affordance brings to light is not only what students *can* do with a technology, but what they are *drawn* to do, the forms of reasoning or shortcut-taking that feel most at hand or natural.

Calculators, for instance, came with a relatively narrow and transparent affordance: the execution of arithmetic, symbolic algebra, calculus, and simulation within a rule-governed symbolic system. Their operations have clear boundaries and often, but not always, easily verifiable outputs. While calculators invite students to offload mechanical computation (Brake, 2024; Haggart, 2023; Hillier, 2023), at the same time they require them to retain some conceptual control. The student must still frame the problem, select the relevant operations, and interpret the results. In input–transformation–output terms, the calculator automates only the transformation stage, the calculation itself, while the input (problem framing) and output (interpretation of the result) remain in the student’s hands. For instance, if a student is asked to split a bill with their friends, a calculator can quickly add and divide the numbers, but it cannot determine which numbers should be added, what represents the total, or why division rather than subtraction is the right operation. The reasoning, which means here translating the problem into a mathematical structure and interpreting the answer in context, remains entirely with the student.

LLMs, by contrast, afford a far broader and more complex set of possibilities. They can generate fluent text on almost any topic, answer any type of question, do research competently in a lot of domains, expand the range of ideas students consider by suggesting multiple perspectives, and even simulate dialogue that resembles a tutoring exchange. These capabilities invite new forms of exploration and creativity, but they also exert new kinds of pressure. Because they can produce polished, plausible, and seemingly complete responses, they also make it increasingly easy for, and inviting to, students to hand over entire chains of reasoning, from brainstorming and structuring to composing and revising. In terms of the input–transformation–output sequence, LLMs can operate across all three stages: they can suggest inputs (reframing the task or prompt), carry out transformations (developing arguments, analyzing data, or generating entire essays), and produce outputs that already appear to contain interpretation and justification.

To take stock, when it comes to affordances, LLMs differ from calculators in three important respects.

● *First*, there is persuasive fluency and the appearance of completeness. LLM outputs are polished, authoritative text, sound, or visuals that seem complete in themselves, whereas a calculator’s bare numbers still demand interpretation before they can function as knowledge. LLMs thus invite students to accept answers as finished products rather than as starting points for further thought.
● *Second* is the scope of automation. A calculator affords the automation only of the transformation stage, that is, the computation itself, while the framing of the problem and the interpretation of the result remain external to the device. A student may still outsource those stages, but not to the tool: they might rely on peers to define the problem or to decide whether the answer makes sense. An LLM, by contrast, can operate at all three stages. It can propose inputs, perform transformations, and generate interpretive outputs, allowing the entire reasoning chain to be offloaded to the same artefact, thus making such offloading easier. This invites students not just to outsource a single step in reasoning, but potentially to hand over the entire cognitive process, from defining the problem to composing the response.
● *Third*, there is uncertainty and its effect on verification costs. With calculators, correctness depends largely on setup; if the inputs are correct, the result will be correct, because its algorithms are deterministic and have been heavily vetted. With LLMs, even well-formed prompts can yield fluent but misleading responses because generation is probabilistic rather than deterministic. So LLMs invite students to trust fluency as a proxy for truth, while placing greater responsibility on them to verify accuracy, coherence, and completeness.

These differences make LLMs’ affordances both richer and riskier (Ali et al., 2023; Sætra, 2023). They broaden the range of cognitive states that can be offloaded, blur the line between knowing and appearing to know, and shift responsibility for verification from algorithmic certainty to human judgment. So, they demand pedagogies that explicitly address these expanded affordances: slowing students down, requiring them to question and revise AI outputs, and cultivating habits of skepticism, interpretation, and reflection. In other words, the educational challenge is not only to integrate LLMs critically into instruction but to help students build the dispositions that keep them reasoning with and about, and not merely through, the machine.

## 5 The need for epistemic virtues

The unique affordance of LLMs, the ability to outsource entire cognitive workflows, presents a distinct pedagogical challenge, as students might be carried away by this technological seduction: they may accept well-phrased but shallow or misleading answers as authoritative, or treat polished text as a substitute for genuine reasoning. In doing so, they risk bypassing the very habits of inquiry, such as questioning, weighing evidence, and revising, that sustain reflective learning. What is needed, therefore, are intellectual dispositions that help students resist this temptation and remain active participants in their interactions with LLMs (Uszkai, 2024). In short, students need specific epistemic virtues, and LLMs designed to foster these virtues.

Epistemic virtues are, essentially, the character traits of a good thinker, and they include truth-conducive cognitive abilities, like critical thinking or open-mindedness, as well as character traits such as intellectual honesty, integrity, courage, humility, and impartiality (Baehr, 2019). As Savulescu (2023) argued, the key epistemic virtues for an age when machines can generate seemingly convincing outputs are the capacity to evaluate reasons, argument and evidence, and to take responsibility for making a decision when preparing a piece of work which, ultimately, has to “stand on the legs of reason”. Others (Naeem, 2025; Hila, 2025; Smith & Vickers, 2024) argue that students have to be taught or steered towards developing curiosity (which motivates them to look more deeply rather than accept easy answers) and carefulness (which makes them thorough and meticulous in assessing LLM outputs), in order to be able to use LLMs constructively. These epistemic virtues could work as mental habits that push back against what LLMs make too easy.

By making fluent, plausible text effortless to produce, LLMs can create a powerful illusion of understanding and completeness (Messeri & Crockett, 2024). This observation is consonant with well-established findings in cognitive science. For example, Sloman and Fernbach (2017) show that people often mistake the availability of information for genuine understanding, a phenomenon they call the “knowledge illusion”. LLMs are able to offer people a lot of information, packaged in an expert-sounding manner, which might amplify their ability to generate “knowledge illusions”. Similarly, research on the illusion of explanatory depth shows that people often feel they understand complex systems far better than they actually do (Rozenblit & Keil, 2002). One reason for this is the confusion between what is internally represented and what can be externally recovered in real time (Rozenblit & Keil, 2002, p. 522): when individuals successfully use a device to solve a problem, they may mistakenly attribute that success to their own understanding rather than to information supplied by the environment. LLMs could potentially amplify this effect. Because they can generate fluent, theory-like explanations on demand, they make it especially easy for users to confuse the model’s readily accessible output with their own grasp of the subject matter.

Thus, the affordances of LLMs call for specific epistemic virtues to help students navigate these challenges. Classical virtues such as humility, carefulness, and open-mindedness remain central to good inquiry, but in an epistemic landscape shaped by LLMs we need to identify which epistemic virtues are best suited to counter the temptations these systems introduce. So, students need virtues tailored to resist LLMs’ temptations: that is, they need to develop a disposition to question confident but ungrounded answers, to slow down rather than accept the first output, and to check sources rather than defer to the system’s apparent authority.

First, patience is necessary as learning often involves accepting ambiguity and partial understanding. Because LLMs produce fluent, authoritative answers instantly, they can make uncertainty feel unnecessary or even uncomfortable, especially insofar as they are used as ‘epistemic authorities’ instead of reasoning partners. Patience helps students resist this temptation, allowing them to remain engaged with incomplete or emerging ideas long enough to refine, question, and deepen them rather than settling for immediate closure.

Second, understanding oftentimes develops through iteration, by revising, testing, and reconsidering ideas. Yet LLMs afford bypassing this step entirely, as students could just input a prompt, receive an answer that sounds convincing and move on. So reflective engagement with ideas would help students think about, revise and question LLM outputs, rather than accepting them at face value.

Third, knowledge depends on evidence and justification, not on how it sounds. LLMs, however, generate text that sounds authoritative and well-structured even when it is inaccurate or shallow. So, students need a healthy dose of skepticism in order to adopt a critical stance toward LLM outputs, reminding them that every claim must be interrogated, checked, and supported before being accepted, even though at first it might seem convincing. (To put it crudely, students must learn to become bullshit-detectors.)

Fourth, knowledge and learning emerge from dialogue, not delegation. Because LLMs can appear all-knowing and they can produce outputs that sound persuasive, they can tempt students to defer uncritically to the machine. Intellectual humility, combined with a critical eye, would then encourage students to approach LLMs as fallible partners in thought, so that they remain open to LLM suggestions but also willing to challenge, refine, or reject them as part of an ongoing exchange.

Our aim here is not to provide an exhaustive list of epistemic virtues for LLMs. This is ultimately a matter that requires the active involvement of educators and educational institutions (Dubljević, 2024). The point is more fundamental. To the extent that LLMs are unlike earlier tools, it is because their affordances make it easier to bypass the very processes of inquiry, drafting, and revision that education is meant to cultivate.

### 5.1 Designing for epistemic virtues

The good news is that the way LLMs are designed and used in education can help support these virtues. Rather than functioning only as content generators, they can be configured to nudge students toward active, reflective engagement with their own thinking, as shown in Table 1. Concretely, this might involve design choices such as:

● **Built-in reflective prompts and feedback loops:** Short questions like “How confident are you really in my answer?” or “Is the evidence I just gave you enough to support this claim?” slow down interaction and prevent students from moving too quickly from output to acceptance. These prompts exercise patience and reflective engagement, asking students to dwell with uncertainty and articulate their own reasons for accepting or rejecting an answer.
● **‘Study modes’ that deliberately withhold closure:** Instead of always producing polished, final-sounding responses, an LLM could offer a mode that provides partial solutions, hints, or counter-questions. Such a mode encourages students to do additional cognitive work themselves, thereby cultivating epistemic patience and resisting the temptation of instant completion.
● **Transparency features: sources, reasoning traces, and confidence levels:** Displaying underlying sources, reasoning chains, or approximate confidence levels makes visible that outputs are fallible and provisional rather than authoritative. This invites intellectual vigilance and carefulness, as students must interrogate the grounds of the model’s claims rather than treating fluency as a proxy for truth.
● **Collaborative critique modes:** Interfaces that encourage students to work together to critique, revise, or improve an AI’s response (for instance by highlighting weaknesses, suggesting alternatives, or weighing competing drafts) could foreground human judgment over mechanical production. These practices support intellectual humility and curiosity, by exposing students to alternative interpretations and to systematic patterns of error in LLM reasoning.

To illustrate how these principles might be put into practice, Table 2 contrasts two ways a teacher might integrate an LLM into the same essay assignment. The first design allows LLMs to be used only for the generation of an answer, giving students little reason to slow down, justify choices, or check claims. The second design uses the LLM as a thinking tool: it requires students to choose between alternative ideas, explain their reasoning, respond to challenges, and verify information.

But, besides cultivating epistemic virtues, policymakers and educators should also consider equity and accessibility. The calculator era demonstrated that equity effects stemmed less from the technology itself than from the pedagogical and institutional capacities surrounding it. A similar pattern will probably also emerge with LLMs. Well-resourced schools are more likely to integrate structured prompting, iterative critique practices, and teacher training that helps students develop epistemic virtues. Under-resourced schools, by contrast, may rely on LLMs primarily for efficiency or error correction, which increases the risk of superficial engagement and epistemic overreliance.

These differences risk widening existing achievement gaps. When students lack access to teacher support or time to engage in reflective, step-by-step learning, they are more likely to accept LLM outputs at face value, rely on fluency as a proxy for correctness, and miss opportunities to practice the virtues that counteract the affordances of LLMs. By contrast, students who have teachers that have been motivated and instructed on how to use LLMs to foster epistemic virtues are better positioned to question confident but unsupported claims, verify sources, and build their own arguments.

What is more, these risks fall disproportionately on marginalised groups, including students from lower-income backgrounds, multilingual learners, and students with disabilities. Such learners often have less access to the resources and instructional conditions required for careful verification, structured practice, and teacher feedback. Equity-focused frameworks from organisations such as UNESCO (Fengchun et al., 2021) and the OECD (OECD Artificial Intelligence Papers, no. 23) similarly emphasise that AI systems in education should “do no harm” to existing inequalities and should be embedded within human-centred, inclusive pedagogical practices. Applying this principle here means recognising that virtue-supportive LLM design is not only a pedagogical question but an accessibility one: without equitable access to the scaffolding that supports epistemic virtues, the benefits of LLMs will be unevenly realised.

In sum, preparing students to engage thoughtfully with LLMs requires a prior commitment to nurturing the necessary epistemic virtues to resist the temptation of passive reliance on automation. From this point of view, humanistic disciplines, such as logic, philosophy, ethics, history or art become crucial in a world where the production of language can be automated but the evaluation of meaning, truth, and value cannot (Suzgun et al., 2025). These domains, more and more sidelined lately, cultivate the habits of mind needed to interrogate sources, question assumptions, and engage with ambiguity, skills that are increasingly essential when fluent, plausible text can be generated instantly, regardless of accuracy or intent.

## 6 Conclusion

In this paper we questioned the increasingly common analogy between calculators and LLMs. The comparison isn’t wrong, exactly: both technologies automate cognitive tasks, both provoke fears about deskilling, and both can support or undermine learning depending on how they’re used. But the analogy is insufficient.

The calculator precedent taught us that outcomes depend on pedagogy, that alignment between instruction and assessment matters, and that equity flows from implementation rather than access alone. These lessons remain valid for LLMs. But our analysis reveals a crucial difference: the affordances of calculators and LLMs fundamentally diverge. Where calculators automate only the transformation stage of problem-solving, leaving framing and interpretation to students, LLMs can operate across all three stages: suggesting inputs, performing transformations, and producing apparently complete outputs. This expanded scope, combined with their persuasive fluency and probabilistic uncertainty, creates new forms of temptation that earlier educational technologies did not.

Our central claim is conceptual rather than prescriptive. We’ve argued that because of their distinctive affordances, LLMs introduce specific epistemic challenges unlike any other technology before: they make it easier to bypass the processes of inquiry, drafting, and revision that learning depends on. They blur the line between knowing something and appearing to know it. They shift verification from algorithmic certainty to human judgment. These features demand something more than good teaching practices. They call for cultivating specific epistemic virtues suited to human-AI interaction.

Education has always aimed to cultivate reflective, critical thinkers. LLMs don’t change that goal. But they do transform what reflection and critique demand. In a world where machines can generate convincing text instantly, the ability to pause, question, verify, and think for yourself isn’t just admirable, it is essential. The question facing educators, designers, and policymakers is not whether to integrate LLMs into education, but how to do so in ways that strengthen rather than circumvent the habits of mind that make learning meaningful. If the calculator era taught us that technology magnifies existing pedagogical strengths and weaknesses, the LLM era requires that we deliberately cultivate the intellectual virtues that automation cannot replace.

## Figures and Tables

**Table 1:** Mapping Epistemic Virtues to Design Features and Pedagogical Practices. This table illustrates how the epistemic virtues discussed in Section 5 might be supported through both LLM design choices and complementary educator practices. The four columns should be read as a connected sequence: each *virtue* (Column 1) addresses a specific *risk* arising from LLM affordances (Column 2); this risk can be mitigated through *design features* embedded in the tool itself (Column 3) and reinforced through *pedagogical practices* in the learning environment (Column 4).

Epistemic Virtue	LLM Risk It Guards Against	Design Feature	Educator Practice
Patience	Instant closure; discomfort with ambiguity	Study modes that withhold full answers; hints instead of solutions	Require outlines or drafts before LLM use; grade process not just product
Reflective Engagement	Accepting outputs at face value	Built-in prompts ("What’s the strongest objection to this?")	Structured critique or editing assignments; use of revision logs
Intellectual Vigilance	Treating fluency as truth	Transparency features: confidence levels, source display, reasoning traces	Fact-checking exercises; compare LLM output to primary sources; compare different LLM outputs
Intellectual Humility	Treating the machine as an epistemic authority rather than a fallible partner	Collaborative modes; multiple competing outputs	Peer review of AI-assisted work; debate formats; red-teaming/devil’s advocate exercises

**Table 2:** Two contrasting ways teachers can integrate LLMs into an essay assignment. This table compares two ways of integrating LLMs into the same essay t’ask: one that allows the model to automate most of the work, and one that deliberately structures the assignment so that LLM use supports epistemic virtues such as patience, reflective engagement, and intellectual vigilance at each stage of the process.

Stage of the task	Automation-driven teacher design	Virtue-supportive teacher design
Setting up the task	The teacher assigns a standard 1500-word essay and gives no guidance on whether or how to use LLMs.	The teacher frames the task as an inquiry: students may use an LLM only to explore ideas, not to generate full essays. Students are told the goal is understanding and reasoning.
Choosing a direction	No planning or checkpoint stage is required, so students often accept the LLM’s first suggestion without reflection.	The teacher requires a short plan or “thinking log” where students explain their chosen angle and how the LLM influenced their decision. This slows the task and prompts justification.
Developing the argument	Rubric emphasises structure and fluency. Lightly edited LLM text can score well, as independent reasoning is not assessed.	Rubric rewards reasoning and judgement (e.g., explaining how LLM suggestions were used, rejected, or modified). Students must write key sections in their own words.
Checking claims and evidence	Teachers don’t warn students of LLM-generated references. Confident-sounding output is rarely challenged.	Teachers explicitly warn about errors and hallucinations; students must check at least one claim or source and briefly record how they verified or corrected the LLM’s output.
After submission	No follow-up discussion. Students can “complete” the task without being able to explain or extend the argument.	A short post-submission discussion, or reflection requires students to articulate their reasoning and describe how the LLM shaped (and sometimes misled) them.

## Data Availability

No data was used for the research described in the article.

## Footnotes

1 By risk we mean the possibility that a tool or practice will undermine a learner’s ability to form true, well-justified beliefs, or will erode important epistemic capacities such as critical reflection or independent reasoning. LLM affordances can generate epistemic risks when they invite users to bypass key stages of inquiry, accept fluent outputs uncritically, or mistake access to generated text for genuine understanding. At the same time, the very same affordances can create epistemic opportunities when they are used to scaffold explanation, broaden the range of ideas considered, or support more reflective, iterative engagement with a topic.

## Data statement

No data was used for the research described in the article.
